# Living with someone else's penis: The lived experiences of two South African penile allograft recipients: A descriptive phenomenological study

**DOI:** 10.1016/j.amsu.2021.102794

**Published:** 2021-09-04

**Authors:** André van der Merwe, Yoesrie Toefy, Mohammed Rafique Moosa, Heidi van Deventer, Chantelle J. Scott

**Affiliations:** a Room 4080, 4th Floor Clinical Building, Division of Urology, Department of Surgical Sciences, Faculty of Medicine and Health Sciences, Francie van Zijl Drive, University of Stellenbosch, Tygerberg, Cape Town, 7505, South Africa; b Division of Community Health, Faculty of Medicine and Health Sciences, University of Stellenbosch, South Africa; c Department of Internal Medicine, Faculty of Medicine and Health Sciences, University of Stellenbosch, South Africa; d Division of Urology, Department of Surgical Sciences, Faculty of Medicine and Health Sciences, University of Stellenbosch, South Africa

**Keywords:** Penile transplantation, Allotransplantation, Vascular composite allograft, Ritual circumcision

## Abstract

**Background:**

In South Africa, penile loss is a recognised complication of ritual circumcision which has a profoundly negative effect on these men's psyches and their everyday lives. The purpose of this paper was to investigate the experiences of the first two South African penile allograft transplantation recipients in order to assess the psychosocial impact of this surgery.

**Materials and methods:**

A qualitative descriptive phenomenology approach was used. A total of four in-depth interviews were conducted with the two South African penile transplant recipients. The interviews were transcribed verbatim while adding the field and observational notes. Thematic analysis was used to derive meaning from the collected data.

**Results:**

After the penile loss, both participants reported feeling suicidal for the following reasons: their communities shunned them and regarded them as ‘dead,’ they felt severely ashamed of their disfigured bodies, they were unable to develop intimate relationships and could not have children. Transplantation gave them a fully functional penis, which resolved the majority of these issues. The participants were able to build relationships and satisfy their own and their partners' sexual and relationship needs. The transplant led to complete restoration of their self-image and manhood. They were, however, still persecuted by their traditional communities as they had not successfully completed the ritual circumcision ceremony.

**Conclusion:**

This study emphasises the necessity of offering penile transplantation as treatment for penile loss as this is not only life enhancing but lifesaving, especially within the South African context.

## Highlights

• Recipients fully accepted the transplanted penis as their own.
• Penile function was fully restored including sexual function and therefore the ability to reproduce.
• Penile transplantation had positive impact on recipients' psychological state and self-image.
• Despite need for lifelong immunosuppressants, recipients were satisfied with decision to have penile tansplant.
• The emotional and social transformation which occurred in the recipients were not only life-enhancing, but lifesaving.

## 1 Introduction

Complete or partial penile loss are recognised complications of ritual circumcision in South Africa, with some 250 men suffering this fate each year [1]; beside penile loss, this ritual also carries a real mortality risk. Penile loss is to cultural purists an indication of weakness and has a significant impact on how these men are perceived by their communities. They are ostracised and are not regarded as ‘men’ in the traditional environment where they are treated as ‘boys’ by men younger than themselves; this is because they failed to complete the ritual circumcision and are left without a functioning penis. While they are perceived as boys, they are treated as if ‘dead’ and not worth including in the affairs of the community. Seeking assistance from Western medicine is considered taboo, adding to the profound impact of penile loss on the psyches of these men [2,3].

The penis is symbolic of masculinity and manhood. It is also an essential organ for sexual pleasure and reproduction [4]. Maslow's hierarchy of needs is a theory that depicts the basic needs of an individual that should be met in order for that person to reach self-actualisation, that is to become the most that he or she can be. These needs are depicted as hierarchical levels with the lowest (and most important) being deficiency needs. The deficiency needs are physiological (food, shelter, sex and reproduction), safety (stability, employment and health), love/belongingness (intimacy, family, sense of connection and being part of a group) and esteem (respect, self-esteem, status, recognition, strength and freedom). The higher-order needs are known as growth needs and entail cognitive and aesthetic needs, self-actualisation and transcendence. This theory states that individuals need to meet deficiency needs in order to be motivated to fulfil growth needs with the end goal of reaching self-actualisation and transcendence [5,6]. For men who are aphallic, many of the mentioned basic deficiency needs remain unmet. According to Maslow, an individual who does not have these basic needs met will be at increased risk of psychological morbidity [7].

Penile loss has been reported to have devastating effects on the psychological state of affected individuals. Studies focusing on the impact of penile amputation after penile cancer have found quantifiable mental illnesses, especially related to feelings of hopelessness, as well as high suicide rates amongst these patients [8,9]. A Norwegian quality-of-life study reported that seven out of thirty patients treated with penile amputation would have preferred a less morbid procedure preserving sexual function even if it meant a reduced lifespan [9]. Significant numbers of severe genital injuries occur in combat. In the Iraqi war, 147 men lost their entire penis and at least one testicle mostly due to blast injuries [10]. As the loss of a penis or other genitalia is associated with a traumatic life event, it often leads to posttraumatic stress disorder. This makes recovery of sexual function more difficult and complex [11,12].

Social death [13], referring to severe psychological withdrawal related to, amongst other things, ostracism [14] and existential suffering, is highly likely present in these men before penile transplantation. Therefore, the question arises whether penile transplantation can reverse this. Existential suffering as defined by Kissane refers to the impact of an advanced medical condition (such as penile amputation) on the individual's life experience and reason for existence [13,15]. Enforced ostracism, as seen in South African men after penile loss, has been labelled ‘the kiss of social death’ [16].

The conventional treatment for penile loss would be reconstructive surgery using own donor tissue such as a forearm fascio-cutaneous flap. Erections for this flap are created by the insertion of a penile prosthesis. While good results are achieved in many cases, complications remain common; these include urethral breakdown and fistulation, flap atrophy that prevents prosthesis insertion, phallic hair growth and prosthesis failure or extrusion. Donor site morbidity is also significant [17]. As previously discussed by [Author], the capital cost of a penile prosthesis approximates US$7330 and there is a relatively high expected complication rate in this patient group, which makes this a less desirable option in the South African context [18]. Although tissue engineering of the penis should be possible one day, current projects to attempt this have been unsuccessful [19].

To our knowledge, no studies that describe the impact of penile loss and penile transplantation from the patient's perspective have been conducted to date. Therefore, the current study aimed at providing a detailed description of the lived experiences of the two South African adult recipients of penile allografts for the treatment of penile loss due to complications related to ritual circumcision. The purpose of sharing these participants' stories is to illustrate the impact that this procedure had on the lives of these men and to argue that this surgery is life enhancing as well as lifesaving.

## 2 Materials and Methods

### 2.1 Approach

A qualitative descriptive phenomenology approach was selected as it allowed the researchers to gain in-depth insight into the phenomena under investigation from the patients' perspective. This was preferred over a quantitative/quality-of-life approach, which would have reduced their experiences to figures and numbers. Maslow's expanded hierarchy of needs as well as the theory of existential suffering as described by Kissane provided the theoretical framework [15].

### 2.2 Procedure

Ethical approval for this study was granted by the Human Research Ethics Committee of our institution. The only two patients who had undergone penile transplant surgery in South Africa were purposively selected and approached by the first author for participation. The primary investigator, AVDM, was the lead surgeon of a team of surgeons who performed the surgery. During a routine follow-up visit, AVDM informed each patient of the aims and objectives of the study and invited them to participate. Informed consent was obtained. They were subsequently contacted by an independent male researcher to schedule a suitable date and time for the interviews.

A male research assistant, YT, was selected to conduct the interviews in order to avoid a power differential effect, since he was not involved in the participants’ medical management. He had also had no prior contact with them. He is an experienced qualitative researcher with a Ph.D in social sciences. His past qualitative studies focused on interviewing men about their circumcision experiences.

The interviews were conducted in a private room at the hospital on a day that the participants had a routine follow-up visit scheduled. YT was the only person present during the interviews. He gained permission to audio-record the interviews, introduced himself, gave a brief background of his research experience and explained the purpose of the study. The initial interviews were conducted over one day per participant and were divided into two sessions of about 4 h each. A topic guide, designed by all the authors, based on the literature and their prior knowledge on the topic, was used to guide the conversation. This consisted of open-ended questions that focused on the participants’ lived experiences of ritual circumcision, penile loss and undergoing penile transplantation and how it impacted their psychological wellbeing, interpersonal relationships and everyday lives. Both participants were contacted for a telephone interview of 20–30 min to clarify further and to add depth to their responses in their initial interviews. All interviews were conducted in English, although the participants were given the option to speak in their mother tongue (isiXhosa), which would have been later transcribed by a translator. A rigid standardised approach was not used when developing the topic guide and conducting the interviews in order to allow the participants to add on to the topics discussed as well as freely express themselves.

The participants also permitted the use of their hospital records to provide further context for the collected data. AVDM and YT furthermore made observational notes about the participants’ body language and demeanour during routine hospital visits and interviews. AVDM is the principal investigator of the main penile transplant study and has been involved in the process of offering penile transplants since 2009. Therefore, he provided further context to the data from his experiences and informal conversations with community leaders, men who had experienced penile loss as well as the recipients. Data collection ceased once the researchers believed that they had collected sufficient in-depth data on each of the cases. As there were only two suitable participants, data saturation was not reached. Additional findings are expected to emerge as more men who have undergone this surgery are interviewed in future.

The interviews were transcribed verbatim by YT, and observational data were added where appropriate. The data were analysed using descriptive thematic analysis. Three authors independently open-coded the transcripts for themes using deductive and inductive methods. Themes were initially pooled around the study objectives to give a detailed description of life before and after penile transplantation. Specific emphasis was placed on how the surgery assisted the individuals in overcoming the existential challenges faced by the penile loss and how they were able to fulfil their basic human needs. The validity of the data was ensured by involving coresearchers in the analysis process. The findings were reported back to the participants for comments to ensure that the data were accurately presented. Atlas.ti7 software was used to assist with data management and the coding process.

No identifying data were collected, and the aliases ‘Thulani’ and ‘Sipho’ were adopted and used throughout the study. This is a vulnerable group, and therefore care was taken to maintain confidentiality at all times. There was a concern that they might be identified by close friends or family as currently there are only two cases. The participants were made aware that psychological support would be available if needed. The work has been reported in line with the Consolidated Criteria for Reporting Qualitative Research (COREQ) checklist [20].

## 3 Results

### 3.1 Participant description

Thulani and Sipho are 43- and 26-year-old males. Both believe in the Xhosa tradition of ritual circumcision. They described being excited and motivated to undergo ritual circumcision as this would have changed their status within their traditional community from a ‘boy’ (as all uncircumcised males of all ages are called) to a ‘man’. Thulani and Sipho attended circumcision school at the ages of 22 and 19 years respectively; the catastrophic result for both was total penile necrosis. Sipho received a penile allograft in 2014, two years after the penile loss. Thulani waited 18 years before he received a penile allograft in 2017.

Sipho was interviewed five years and Thulani about two years after the allotransplants. Despite being interviewed years afterwards, both men still became emotional when recalling the traditional circumcision event neither participant successfully completed. As a result, they were persecuted and ostracised by their communities. They feared to talk about their ordeal as they feared that they would be harmed by cultural purists. They refrained from using public spaces, especially restrooms, as they had to sit when urinating. As a result, they became socially isolated and both decided to relocate.

Sipho had supportive sisters and relocated to a different province to live with them. Thulani did not have the same level of support and experienced rejection from his family. His brother had been the person who was delegated to ensure his wellbeing during circumcision school; he partly blames his brother for what happened. Thulani already had a strained relationship with his father that was further tested by the ordeal; he therefore did not have the needed support from his family to deal with his trauma. He decided to protect his secret at all costs and isolated himself socially. He dropped out of school and started moving around South Africa to avoid building close relationships. He chose areas that had few resident Xhosa individuals and relocated as soon as a Xhosa person moved in. He felt that this was necessary because, in their culture, it is completely acceptable to ask members to prove a successful circumcision by a show of genitalia. A ritual circumcision leaves a typical scar that appears different from the fine surgical suture line of medical circumcision. As a result of their relocation and psychological distress, both men were unable to maintain a steady job, build a career or maintain friendships.

Thulani had an understanding and supportive intimate partner before losing his penis. Their relationship lasted for a few years after the loss before he felt he had to ‘let her go’ because he could not satisfy her sexual needs or give her children. Subsequently, he was unable to form other meaningful relationships; he would be in a relationship for a short period and then fabricate excuses to break the friendship before the relationship became sexual. Sipho ended the relationship that he was in shortly after returning from circumcision school and having a penile amputation. He did this as he did not feel that this relationship was serious enough to justify sharing this sensitive information. After this relationship, he struggled to maintain new relationships. Thulani describes the period between losing his penis and receiving the transplant as a devastating and depressing period. Sipho shared this sentiment and added that he would prefer to forget about it again as the problem had been resolved.

During informed consent for the transplant, both were informed of the risks of immunosuppression and were offered phalloplasty. In public hospitals in South Africa, penile prosthesis is not an option due to the cost.

### 3.2 Themes

#### 3.2.1 Penile transplant surgery and recovery

Both men declined phalloplasty as they felt that the only viable solution for them would be a ‘real penis’. Thulani explained his reason for declining this option: “I still want sex, I still want kids and what he is offering me is not going to give me those things, so let's just forget about it. And as I was about to leave, a certain doctor told me: listen, there is a Professor [academic hospital], he is doing this kind of research about doing a penis transplant. It's going to be fully functional, but I am going to be on medication for the rest of my life. A medication that might result in a cancer in about ten years' time. And I told him that I will make two babies before I develop the cancer.”

Both Thulani and Sipho successfully received penile allotransplants. They were initially in disbelief that it had happened and were both anxious and excited to see what their penis looked like. During the first removal of bandages, Sipho, had tears in his eyes and was silent for a few minutes. He was elated with the results and described feeling ‘number one’. Thulani, however, was not pleased by what he initially saw. When asked to elaborate, he explained, “Because it was swollen and scary with all the stitches, and I was like: My God, what kind of a penis is this? As time went on, it improved.”

After surgery, Sipho and Thulani remained in the hospital for a month. The penises gained full function, and at three weeks, they were experiencing unpleasantly painful erections as the scars were still healing. They were prescribed immunosuppressive medication that had some bothersome side effects. Each had to take a large number of tablets every day, and frequent hospital visits were required. Multiple blood samples were taken to assess blood levels to establish the correct drug dosages and monitor for adverse effects. Both participants had skin rashes. Sipho also had swelling in his extremities due to a serious fungal infection. These side effects seemed to bother Sipho more as he was a competitive bodybuilder. He stopped taking his medication about two years and eight months after surgery as the skin rash negatively affected the outcome of the competitions. He lost about half of his allograft before transplant rejection was successfully reversed, but he had to have skin grafts.

Despite these complications and the need for chronic medication, the participants expressed gratitude for the penile allograft. Even though both participants struggled with taking the medication initially, it became part of their daily routine. They will happily continue taking their medication, despite the potential side effects, as the alternative is worse. They do not ever want to go through the trauma of losing their penis again. Thulani stated, “Yes, at first, it [taking the medication] was a bit difficult because I wasn't used to, but for me that is the easiest part. You see, because this is something I am not willing to lose. Not again. So if I want to keep this, then I must continue enjoying my meds.”

#### 3.2.2 Living with someone else's penis

Thulani received a circumcised donor penis that was a poor skin colour match. However, this did not seem to be an issue for him: “They told me before the operation that the donor was a white person and was I going to be comfortable with the donor being a white person? I told the sister: Sister, I want a penis. That's what I want and I am not going to take my pants off and run all over [township] to show them my penis. So I don't care if it is white, or it's pink or purple. I will settle for anything.” Life without a functional penis was devastating to the degree that he was content with whatever colour penis he was given, provided that it was functional.

The offer was made to Thulani to have the graft penis tattooed to match his skin colour, to which he responded, “I don't know if I'm okay with it, I'm so used to this one, it will take some time to get used to a new one [laughing]. I was talking to a friend and I said to him: You must not think we are the same? I'm special and you are not. And he was like: ‘What makes you so special?’ I said that I am the only Black man in the whole wide world with a white penis [laughing]. So yeah, but one of the other reasons we decided not to dye it was that Prof was afraid that I would get an infection.” Thulani has fully accepted his penis and feels that it is something that makes him unique. He has also found a way of explaining it to a female partner or if anyone asks by telling the person that he has vitiligo, a common disease amongst black South Africans.

In contrast, Sipho received an uncircumcised donor penis that was a good colour match. For this reason, he feels that it looks similar to the one he lost. He is also not bothered about what it looks like as, once again, the functionality of the penis is more important.

Both participants admitted to sometimes being aware that they have someone else's penis attached to their bodies, but then they quickly remind themselves that it is now theirs and part of their body. Thulani further rationalised that the donor had died and had no further use for his penis.

#### 3.2.3 Reintegration into society

Both recipients described feeling less socially isolated and more confident after the transplant. They felt more comfortable in social spaces and were able to build new friendships. They were able to use public restrooms without the fear of people finding out what had happened as they were able to urinate standing. This was more so for Sipho as he had received a penis that was a good colour match.

The participants emphasised that they had been ‘brought back to life’ by undergoing transplantation. Sipho explained that he had chosen to have this surgery as he ‘chose life’. In the Xhosa culture, aphallic circumcision failures are seen as ‘dead’ as they cannot perform their ‘duties’. A community leader explained that by giving these men a penis it would be like ‘bringing them back to life’. Thulani and Sipho felt that by having this surgery, they were able to reintegrate into their social communities; however, full acceptance by their Xhosa peers was not as simple. Thulani explained, “At some point I thought, okay, if we do this transplant and it works, people would look at things differently and see a second chance, not only for me but for others too, you know? But then again, it's not happening … Of which is something I told myself that I wasn't going to bother myself much with. Because … at the end of the day, it's not about people and their negative thoughts, it's about me and I have made peace with the fact that they will never fully accept us.”

If found out, these men will still be seen as boys and will not be accepted by the community and be ostracised again. As a result, they remain fearful of disclosing what has happened. Despite this persecution, the participants decided to rather focus on the inner peace that they had found and the positive impact the transplants had on their personal lives.

#### 3.2.4 Intimacy and sex

Both participants described a vast improvement in their ability to have intimate relationships and were excited to be having sexual intercourse again. Thulani explained, “I was told not to rush things. You know, wait for it to heal properly before I can go and paint my areas sexually. [laughs]. So yeah, that's what I did, and I was excited. That's one feeling I would never forget [having sex again].” After many years of avoiding relationships, Thulani successfully entered into a sexual relationship shortly after his transplantation. They dated for a few years before the relationship ended. He described being confident in his ability to satisfy his partner's needs.

Sipho remained single after his penile loss. He entered into a relationship shortly before having the penis allotransplant. He described his girlfriend as being very understanding of his situation. He did, however, feel the need to have sex with her as soon as he recovered from surgery. “Yes and then after I was going out [discharged, five weeks after the penis transplant] … the same day I was do the sex.” He impregnated his girlfriend, but sadly their child was stillborn at term and this lead to the end of their relationship. After which, Sipho became promiscuous, having sex multiple times per day and having multiple sexual partners. Despite advice to use condoms, Sipho contracted urethritis that was treated successfully.

Both participants described the sensations during sex as being the same as with their native organs. Both felt this was a major positive aspect of receiving a penis transplant as they were able to enjoy something that they had been deprived of for many years. For them, it was not only about the sexual act but also about their ability to build lasting relationships, which is a necessity when trying to lead a fulfilled life. Regaining their normal sexual function seemed to have spearheaded their psychosocial recovery and has led to a major positive change in their mood and wellbeing.

#### 3.2.5 Restoration of self-image

The penile loss had devastating effects on both participants’ psychological state, especially with regard to self-esteem and identity. They described feeling severely depressed and at times harbouring suicidal thoughts. As they had lost the essence of their masculinity and manhood, they felt cast out, worthless and ashamed. Thulani found it difficult to look at himself in the mirror and see his maimed body. At times, he did not wash the lower part of his body as it was emotionally too painful. He resorted to heavy drinking as a coping mechanism. Post surgery, he felt like a different person and looking at his lower body was more pleasing. When asked what it meant to receive a penile transplant, Sipho responded, “It is good to be a man again!” Both appreciated the restoration of their manhood. They now put effort into their grooming, dress smartly and seem more confident and joyful.

The participants described that the transplant had given them hope and motivated them to lead a more meaningful life. An important aspect of leading a meaningful life that was restored was their ability to reproduce. Having children is important for Xhosa families as their wealth is measured by the number of children that they have. Sipho explained, “Yes it changed my life, because of if I didn't get the penis back maybe I don't [have children], we want our children.” Thulani felt that not being able to have children was one of the biggest losses that he experienced after having a penile amputation. The fact that he was able to impregnate his girlfriend, has restored his hope to have children.

Both participants stated that they would accept the transplant again, due to the positive impact that it had on their lives. Thulani explained, “It made me look at life differently. For instance, there were times where I didn't see a point in living, you know? But now I see myself as being lucky that I'm still here … and alive [not committing suicide]. Because there are people who did not get the chance. I still want to live some more …. There are certain things I still want to do. I wish I can help where I can. The reason for that is that I also want to change lives. I mean, I got lucky. I met people who managed to change mine. For instance, one of the positive things … I used to drink heavily … That's why I am saying that I look at life differently, you know? I sort of look at the bigger picture. The blinkers that were there before are no longer there. I look around and I look at the possibilities.” Having a penile transplant cured the participants of their unhealthy coping strategies and enabled them to pursue life goals that they had previously neglected including finding a stable job.

## 4 Discussion

Qualitative data in vascularised composite tissue allotransplantation are sparse. Herrington, reporting on five hand transplant cases, found a great improvement in patients’ overall health and recommended that a qualitative component be added to all such cases [21]. Our qualitative study provides an interesting insight into the real-life experience of two men who received a penile transplant. Receiving a penile transplant was experienced as daunting but exciting at the same time.

At first sight, the penis was not entirely what was expected, but after the recovery period, the penis was aesthetically pleasing and fully met the expectations of the recipients. They accepted and integrated the organ fully as their own. This is in contrast with a previous case reported from China where a penile transplant was attempted in 2005. The transplanted penis had to be removed in the early postoperative period due to ‘psychological rejection’. Photographs of the Chinese transplant showed a partially necrotic-appearing penis. This might have added to the psychological rejection [22,23].

Psychological rejection in renal and cardiac transplantation is well described and might relate to the altered self-image with the introjection of a new organ. It is postulated that the self-image becomes fluid again and that the inserted organ causes a ripple effect on the self-image that might even induce psychosis [24,25]. The reason our two cases and the two successful American cases [26,27] have not shown psychological rejection might relate to careful case selection, adequate psychological preparation preoperatively, the small sample size or perhaps the relief of existential suffering and ‘social death’. Existential suffering and social death were likely present due to the circumstances of the ostracised recipients fearing for their lives before the penile transplant.

Major forms of existential suffering that are prevalent amongst individuals who have a failed ritual circumcision include death anxiety, major change, loss of control, fundamental aloneness and altered quality of relationships. The rejection and ostracism experienced from their traditional communities, losing their partners, and in Thulani's case, losing his family support, likely enhanced their existential suffering and their social death. This might have created a strong enough need to overshadow the potential psychological rejection of the transplanted organ [15].

Before transplantation, social death was most likely present in both recipients. While both our cases were (and to a lesser degree still are) in physical danger from cultural purists as they ‘failed the custom’, they were most likely suffering from a psychological form of social death before receiving their transplants. While the medical form of social death is irreversible, the social science model is a reversible multifaceted one, comprising social isolation, loneliness, ostracism, change in personhood, change in role and identity, harm and disfigurement [13].

Psychological social death and existential suffering in the absence of psychological support are likely to have induced the suicidal thoughts in both participants. In contrast, with the two American cases, less shame and more support were present before their penile transplants [26,27]. In the first case, the recipient lost his penis after penile cancer surgery. The second case was a war veteran who lost his penis, part of his scrotum and lower abdomen after a blast injury in the line of duty. He would always be respected as an American hero and be completely accepted by his community while aphallic. Compare this to the South African context where the penises were transplanted in the utmost secrecy, the hospital allocated fake names and the staff were trained to avoid possible media leaks to prevent cultural purists or the recipients’ community from harming them. Until now, the participants have both refused any type of media interview and insisted that the recordings of the interviews for this study be deleted to ensure confidentiality.

The penile transplants led not only to a physical transformation but also to an improvement in the recipients' psyches. Their self-worth was restored, and they were ‘brought back to life’ from social death. Therefore, by choosing to have penile transplantation and all that it involved, as explained during the informed consent process, they actively “chose life”, in the words of Sipho. Penile transplantation allowed them to become ‘men’ again as they were transformed from being aphallic to phallic, which made their bodies feel whole again. They were given back their symbol of manhood and could once again look at themselves with pride. Their confidence was restored, which was evident in their change of demeanour and appearance.

After transplantation, the men were able to become active members of their new communities. They stopped hiding, despite the danger of not being accepted by their traditional community, should they be found out. They felt more comfortable in public as the presence of a penis, and not having to sit down to urinate, made it easier to hide their failed ritual circumcision. Interestingly, after the period of immunological rejection and partial loss of Sipho's penis, the penis has the appearance of being partially mutilated by ritual circumcision.

Both participants were able to maintain new long-term relationships and regained their full sexual function. Erection-enhancing oral medication was not needed by Sipho and was occasionally needed by Thulani towards the end of 2019. They were able to satisfy their own sexual needs, as well as those of their partners, describing sexual intercourse as being as pleasurable as before their penile loss. In earlier interviews, Sipho claimed that he was sexually active up to, or more than, five times per day over weekends. It is unlikely that the same result would be achieved by skin flap phalloplasty and an inflatable prosthesis, which might extrude at these high levels of sexual activity.

One of the most significant outcomes was that the transplants enabled the recipients to have children. Even though only Sipho impregnated his partner, the potential for having children is also very important to Thulani. This is interesting as during the counselling process of informed consent, they were informed of assisted reproduction that would enable them to have children should they not have a penile transplant. Assisted reproduction is expensive in South Africa as even state patients have to pay full private rates. This might have caused them to conclude that it was impossible due to financial constraints. Alternatively, ‘selective hearing’, often guarded against in an informed consent process of innovative treatments and vulnerable groups in life-or-death situations, might have accounted for this finding [31].

The men regained most of the self-identity and self-worth that they had had before their ritual circumcision ordeal. They seemed to be positive about their futures and felt more confident in trying to build a proper career for themselves to better provide for their future partner and children. They described wanting to live life to the fullest and to try all the things that they were previously unable to do. This experience is similar to that reported by heart, liver and kidney transplant recipients for whom potential existential suffering was reversed [29,30].

The identity restoration of these men may be compared to the experiences of women receiving breast reconstruction after mastectomy for cancer to restore their womanhood and their sexual desirability [28]. Losing an organ unique to a man or a woman has a devastating impact on individuals’ sexual identity, and restoring their disfigured bodies restores their gender identity and their self-confidence; it facilitates acceptance by their communities and aids in building relationships. This is essential for living healthy, meaningful and fulfilled lives.

From the evidence provided, it is clear that penile transplantation addressed many of the basic needs described at each level of the hierarchy proposed by Maslow [6]. The respondents’ physiological needs of sex and reproduction, safety needs of employment and stability and needs of love and belongingness were fulfilled, and their self-esteem was restored. As they were able to satisfy these needs, they now have the motivation to reach self-actualisation.

Despite the overall positive impact that the transplants had, it needs to be weighed against the recipients’ need for lifelong immunosuppressants with potentially serious adverse effects. Despite the latter, the participants both felt they would gladly continue taking the medication as the alternative of being aphallic or having a phallopasty was not desired.

The question remains whether phalloplasty with penile prosthesis would have produced similar results in terms of sexuality and restoration of the self, but without the need for immunosuppression. The literature is scant on nontransgender male phalloplasty satisfaction reports. However, Callens et al. [32] investigated 18 males who had received phalloplasty; all cases had had multiple operations. Most respondents (10 out of 18) seemed sexually active at least via masturbation. However, 75% reported being inhibited in engaging in consensual sexual activity. Although 90% reported overall satisfaction, 50% were unhappy with the appearance of the genitalia. All five men in whom a urethral reconstruction had been attempted had fistulation, and three developed urethral strictures. Three out of five patients with attempted urethral reconstruction could stand to urinate. The men in the traumatic penile loss subgroup were the least satisfied with the results. Extrapolation of these reports from Belgium to Sipho and Thulani's context, where the penile prosthesis was not an option, is difficult.

The drive behind penile transplantation in South Africa was to find a near-perfect, cost-effective solution for ritual circumcision-related penile loss that allowed restoration of the normal functions of the penis. As generic immunosuppression medication has become affordable, donor defects are avoided, sexual and psychological restoration results appear to be near perfect and the current side effects of immunosuppression do not seem to have a major effect on the two South African cases (and the two successful American cases), penile transplantation is probably the current gold standard for reconstructing major genital defects. However, in an informed consent process, phalloplasty should always be offered as this option excludes potential toxic immunosuppression.

The limitations of the study are the small sample size and recall bias due to the long period since the transplantations. In interviews that [the first author] conducted with both participants, during the main study from 2011 to 2014, suicide was a more prominent theme in comparison to the themes arising from the latest interviews, even with prompting. We plan to do qualitative research on subjects who have lost their penis, but have not had a penile transplant to gain further clarity on why this discrepancy was observed. The sensitive nature of the topic may have caused the participants to withhold certain details of their experience that may be too traumatic to share. Sipho was not as forthcoming with information despite both participants being offered the services of an interpreter. This could have caused a skewing of the information to a degree because most of the data arose from Thulani. Despite these limitations, the study finds strength in the novelty of the research topic. The participants had a unique story to share, and this study provides the first in-depth account of how penis transplants impact the lives of the recipients. This is important information when offering this surgery to others.

## 5 Conclusions

Penile transplantation gave men in this study a second chance in life due to the restoration of their self-image and sexuality. It had a major positive impact on their psychological state and everyday life. The study provides evidence that an emotional and social transformation occurred after receiving a new penis and that these men can meet the basic needs that all people aim to fulfil. The surgery is not only about restoring the ability to have sex for these men, but also about restoring their self-esteem and ability to have children. This provides them with the confidence to pursue self-actualisation and to lead meaningful lives. From their perspective, the surgery was lifesaving, not merely life enhancing.

## Provenence and peer review

Not commissioned, externally peer-reviewed.

## Ethical approval

Ethical approval granted by the Health Research Ethics Committee of the University of Stellenbosch. Reference number is N11/07/243.

## Sources of funding

Internal funds from the department of Urology at the University of Stellenbosch were used to fund this study. There were no external funders.

## Author contribution

**Andre van der Merwe:** Conceptualization, Methodology, Investigation, Formal analysis, Data curation and Writing: Original Draft, Review and Editing.

**Yoesrie Toefy:** Investigation, Data curation, Writing: Review and Editing.

**Mohammed Rafique Moosa:** Conceptualization, Supervision, Validation and Writing: Review and Editing.

**Heidi van Deventer:** Writing: Original Draft, Review and Editing.

**Chantelle Jennifer Scott:** Supervision, Methodology, Formal analysis, Investigation, Validation and Writing: Original Draft, Review and Editing.

## Registration of research studies

1 Name of the registry:Clinical trials: Penile Transplant Study.
2 Unique Identifying number or registration ID:NCT03149692.
3 Hyperlink to your specific registration (must be publicly accessible and will be checked):https://www.clinicaltrials.gov/ct2/show/NCT03149692.

## Guarantor

Prof Andre van der Merwe.

## Consent

Written informed consent was obtained by both participants. The consent forms were in Xhosa as this is the first language of the participants. We also have English copies which is available upon request.

## Data statement

The interview topic-guides, anonymised interview transcripts as well as the Atlas.ti outputs are available on request. The audio-recordings of the interviews have however been destroyed as requested by the participants in order to protect their identity.

## Declaration of competing interest

None for any of the authors.
